# Effects of the levonorgestrel-containing intrauterine device, copper intrauterine device, and levonorgestrel-containing oral contraceptive on susceptibility of immune cells from cervix, endometrium and blood to HIV-1 fusion measured *ex vivo*

**DOI:** 10.1371/journal.pone.0221181

**Published:** 2019-08-22

**Authors:** Marielle Cavrois, Joan F. Hilton, Nadia R. Roan, Margaret Takeda, Dominika Seidman, Sarah Averbach, Eric Chang, Nandhini Raman, Ruth Greenblatt, Barbara L. Shacklett, Karen Smith-McCune

**Affiliations:** 1 Gladstone Institute of Virology and Immunology, San Francisco, California, United States of America; 2 Department of Epidemiology and Biostatistics, University of California San Francisco, San Francisco, California, United States of America; 3 Department of Urology, University of California San Francisco, San Francisco, California, United States of America; 4 Department of Obstetrics, Gynecology and Reproductive Sciences, University of California San Francisco, San Francisco, California, United States of America; 5 Department of Obstetrics, Gynecology and Reproductive Sciences, University of California San Diego, San Diego, California, United States of America; 6 Departments of Clinical Pharmacy and Medicine, University of California San Francisco, San Francisco, California, United States of America; 7 Department of Medical Microbiology and Immunology, School of Medicine, University of California Davis, Davis, California, United States of America; CEA, FRANCE

## Abstract

Globally, HIV/AIDS is a leading cause of morbidity worldwide among reproductive-aged cisgender women, highlighting the importance of understanding effects of contraceptives on HIV-1 risk. Some observational studies suggest there may be an increased risk of HIV-1 acquisition among women using the long-acting injectable progestin contraceptive, depo-medroxyprogesterone acetate. The potential mechanism of this susceptibility is unclear. There are few data on the role of the upper female reproductive tract in HIV-1 transmission, and the mechanisms of HIV-1 infection are likely to differ in the upper compared to the lower reproductive tract due to differences in tissue composition and variable effects of sex steroids on mucosal immune cell distribution and activity. In this study, we measured the susceptibility of mucosal immune cells from the upper female reproductive tract to HIV-1 entry using the virion-based HIV-1 fusion assay in samples from healthy female volunteers. We studied 37 infectious molecular clones for their ability to fuse to cells from endometrial biopsies in three participants and found that subtype (B or C) and origin of the virus (transmitted founder or chronic control) had little influence on HIV-1 fusion susceptibility. We studied the effect of contraceptives on HIV-1 susceptibility of immune cells from the cervix, endometrium and peripheral blood by comparing fusion susceptibility in four groups: users of the copper intrauterine device (IUD), levonorgestrel-containing oral contraceptive, levonorgestrel-containing IUD and unexposed controls (n = 58 participants). None of the contraceptives was associated with higher rates of HIV-1 entry into female reproductive tract cells compared to control samples from the mid-luteal phase.

## Introduction

An estimated 14.3% of women of reproductive age use intrauterine devices (IUDs) globally [1]. However, little is known about the impact of IUD use on mucosal immunity of the female reproductive tract, and whether it influences risk of HIV-1 infection. Most literature on HIV-1 risk in IUD users was published mainly in the 1990s and focused on the copper IUD, before the now commonly used levonorgestrel (LNG)-containing IUD was widely available. In 2007 and 2012, the World Health Organization (WHO) convened technical panels to discuss hormonal contraceptives, IUD use and HIV-1 risk [2, 3]. They concluded that none of the existing prospective studies found an association between IUD use and HIV-1 acquisition, but the numbers of studies, and of observations of IUD-users, were small [4–6]. The available cross-sectional studies were mainly focused on the copper IUD and were limited by methodological issues such as failure to control for confounding factors, and unclear timing between IUD use and HIV-1 acquisition [2]. The panel concluded: “Current evidence suggests that the use of the copper IUD does not increase the risk of HIV-1 acquisition. However, this evidence is limited and weak.”[2] The panels also concluded that most available research assessed hormonal contraceptives or progestin-only injectable contraceptives such as depo-medroxyprogesterone acetate, whereas there is little evidence about the potential relationship between HIV-1 risk and other contraceptive methods such as IUDs. The 2012 panel stressed the need for ongoing research to evaluate the effects of hormonal contraceptives on HIV-1 acquisition risk [7].

Understanding the effects of contraceptives on HIV-1 acquisition is essential given that HIV/AIDS is a leading cause of morbidity and mortality in women in their reproductive years [8]. In addition, observational studies suggest an increased risk of HIV-1 acquisition among women using hormonal contraceptives, specifically the long-acting injectable progestin contraceptive, depo-medroxyprogesterone acetate [9]. A recent randomized trial compared rates of HIV acquisition among women using depo-medroxyprogesterone acetate, a copper IUD and a levonorgestrel implant, and showed no significant differences in HIV risk between the groups; these results are reassuring about the safety of each of these methods [10]. This trial however did not study oral contraceptives or the LNG-IUD, as was done in this study.

There are few data on the risk of HIV-1 acquisition relating to upper female reproductive tract (FRT), which includes the endocervix and endometrium. The mechanisms of HIV-1 infection are likely to differ in the upper compared to the lower FRT due to cyclic effects of sex hormones on relevant characteristics of mucosal immunity [11–14]. Additionally, the upper FRT is lined by a single layer of columnar epithelium which is more susceptible to injury and absorption of exogenous substances than the vagina and ectocervix, which are lined with a multi-layered squamous epithelium that functions effectively as a barrier to systemic access. The parallels between the immunological characteristics of the upper FRT and the gastrointestinal tract highlight the importance of studying the upper FRT as a portal of HIV-1 acquisition [12]. Indeed, studies in primates confirm that SIV infection can occur in the upper FRT [15].

We previously reported that the LNG-IUD created both inflammatory and immunosuppressive changes in the mucosal microenvironment in the upper FRT [16]. Samples from the endometrium and endocervix of women using the LNG-IUD showed higher proportions of CD4^+^ T-cells expressing both CXCR4 and CCR5, the two main HIV-1 coreceptors, compared to controls. Activated CD4^+^ T-cells (i.e. CD4^+^CD38^+^HLA-DR^+^ cells) were also more abundant in endometrial samples from LNG-IUD users than controls. These phenotyping experiments suggested that the upper FRT of LNG-IUD users may be more sensitive to HIV-1 acquisition as compared to non-users. However, in that prior study we did not test susceptibility of FRT cells to HIV-1 infection. Furthermore, given that the LNG-IUD combines a hormonal treatment (levonorgestrel) with a device (the IUD), the effects observed in our previous study could result from either or both interventions. In this study, we compared immune cells of women exposed to levonorgestrel systemically using LNG-based combined oral contraceptives (COCs), locally using the LNG-IUD, to a hormonally inert IUD (copper IUD), or to neither IUD nor hormone, for their susceptibilities to HIV-1 entry using the virion fusion assay. We chose the following anatomic sites as sources of immune cells to study HIV-1 susceptibility: peripheral blood, as it is a commonly used reference source of cells for HIV-1 infection studies; the cervical transformation zone, as it constitutes the junction between the upper and lower reproductive tracts, is enriched for immune cells [17] and is the site at which HIV-contaminated semen would first make contact with the upper FRT; and the endometrium, as it is the primary site of contraceptive effects. Using samples from these sites obtained from healthy female participants, we measured *ex vivo* the HIV-1 fusion susceptibility of immune cells as a surrogate marker for the potential risk of HIV-1 susceptibility in women using these contraceptives.

## Materials and methods

### Study design

This cross-sectional study compares HIV-1 fusion to immune cells from the blood, endometrium and cervix from samples donated by 4 groups of HIV-negative women: women using no hormonal or intrauterine contraception (controls), women using copper IUDs, women using LNG-IUDs, and women using LNG-containing COCs. The UCSF Human Research Protection Program & IRB approved the study protocol, recruiting and consent materials.

### Recruitment and screening of human volunteers

Healthy women volunteers age 18–45 years from San Francisco and the greater Bay Area were recruited via flyers placed in a variety of venues, local publications, and social media. Volunteers were pre-screened by telephone to ensure they were eligible for the control group or were using one of the designated methods of contraception for the past 6–48 months, with a goal of recruiting an equal number of women per group. Participants in the COC group were included if they were using a 28-day pill pack of combined contraceptive (estrogen plus LNG) containing either 0.10 or 0.15 mg of LNG per tablet on a cyclic schedule; participants taking the pills continuously were excluded. Participants in the control and copper IUD groups were included if they had regular periods every 21–35 days. Potential participants were ineligible if they had undergone hysterectomy, were breast-feeding, were within 6 months of parturition, had abnormal cervical cytology in the past year, used systemic corticosteroids or immune-modulating therapies or used non-steroidal anti-inflammatory agents daily, were unwilling/unable to refrain from vaginal intercourse for 3 days prior to specimen collection, or were unwilling to use non-lubricated condoms throughout the duration of the study. Candidates were scheduled for a screening visit, at which time study personnel explained procedures in detail; obtained written informed consent and demographic information; collected urine to test for pregnancy, *Chlamydia trachomatis* and *Neisseria gonorrheae*, and collected blood for HIV-1 serology. Candidates were ineligible if they had a positive result on any of those tests or had clinical evidence of vaginitis, vaginosis or pelvic inflammatory disease.

### Sample collection

Participants were taught how to use urine testing kits for detection of luteinizing hormone (LH) (ClearBlue Ovulation test Digital, Proctor and Gamble, Cincinnati, OH) and were asked to phone study personnel when the test showed a positive result. Women in the control and copper IUD groups were asked to present for biopsies 7 to 11 days after a positive home urine LH test. Women using LNG-IUDs were asked to present for biopsies 7 to 11 days after a positive home urine LH test or at their convenience after testing for 2 months with no positive result, whichever came first. COC users were asked to present for biopsies on day 12–16 of their pill pack. All participants had a blood sample collected for measurement of plasma progesterone level on the day of biopsy, and in the COC and LNG-IUD groups, for measurement of LNG levels. All participants were confirmed to have a negative urine pregnancy test on the day of biopsy.

For sample collection, a speculum was inserted into the vagina, the cervix was visualized and the posterior vaginal fornix was swabbed with a Q-tip for determination of pH on pH paper (VWR, Visalia, CA) and a second Q-tip for measurement prostate specific antigen (Abacus Diagnostics, West Hills, CA), a marker of recent vaginal intercourse. If cervicitis or vaginitis was noted, a wet mount was performed, and the specimen collection visit was canceled if bacterial vaginosis, candidiasis or trichomoniasis was diagnosed. Blood was obtained for isolation of peripheral blood mononuclear cells and for measurement of progesterone (Quest Diagnostics, West Hill, CA) and levonorgestrel (University of Southern California Reproductive Endocrinology Laboratory, Los Angeles, CA). A speculum was inserted into the vagina and the cervix was washed with Lugol’s iodine solution and an endometrial biopsy was obtained with a 3 mm biopsy cannula (Miltex brand Softflex) inserted through the cervical os. If necessary, the ectocervix was injected with 1% lidocaine and a tenaculum was placed for retraction. If the amount of endometrial tissue was assessed to be inadequate, a second pass of the cannula was made. The cervical transformation zone (TZ) was identified as the junction between the Lugol’s staining and non-staining epithelium and 2 biopsies at separate locations were taken using a Tischler biopsy forceps; if the TZ could not be identified because the entire ectocervix was stained, the biopsies were taken with one of the biopsy prongs inside the os. Some participants could not provide biopsies from both anatomical sites (endometrium and cervix) due to intolerance of the procedure and some participants were unable to tolerate phlebotomy. Of 58 participants studied, 40 (69%) donated samples from all three sites (endometrium, cervix and blood), 17 (29%) from two sites and 1 (2%) from the cervix only.

### Preparation of single cell suspensions

Sample preparation, the HIV-1 fusion assay, and FACS analysis were conducted by a researcher blinded as to the group assignment of the women who donated the samples. Endometrial and cervical biopsies were placed in a 15 ml centrifuge tube containing 5 ml of RPMI supplemented with 2% fetal bovine serum and were processed and frozen within 2–3 h of collection. When needed, the cervical biopsies were cut into smaller pieces (2 x 2 mm). To generate single cell suspensions, the biopsies were first centrifuged at 365 x g for 5 min. The supernatant was removed by aspiration and the sample resuspended in 1 ml of phosphate buffered saline. After addition of 1 ml of the 2 X digestion buffer containing collagenase Type I, hyaluronidase, and penicillin/streptomycin as described [18]; digestion was allowed to proceed for 1.5 h at 37°C. On a few occasions, when tissue pieces were small, the digestion was stopped earlier when the entire tissue was visibly digested. The cells were washed, pelleted and frozen in 1ml of fetal bovine serum containing 10% dimethylsulfoxide. An aliquot was taken from the freshly digested suspension and stained with an immunostaining panel that included an anti-CD45 antibody conjugated to APC (BD biosciences, reference 555485) and anti-CD235a conjugated to FITC (BD biosciences, reference 559943), a marker of red blood cells (RBCs). The ratio of RBCs to white blood cells (WBCs) in blood is typically 700:1, and we excluded samples with >7000 RBC per WBC as those samples had high levels of blood contamination (≥10%). This resulted in exclusion of one endometrial and 2 cervical biopsies. Samples with total numbers of white blood cells < 200 were also excluded. The median yield of white blood cells from endometrial biopsies was 1.3 x 10^6^ (range 0.033–47 x 10^6^), from cervical transformation zone biopsies was 110,000 (range 0.005–4.2 x 10^6^) and from blood was 2.4 x10^6^ (range 0.3–26 x 10^6^).

### Fusion assay

**Fig 1:**
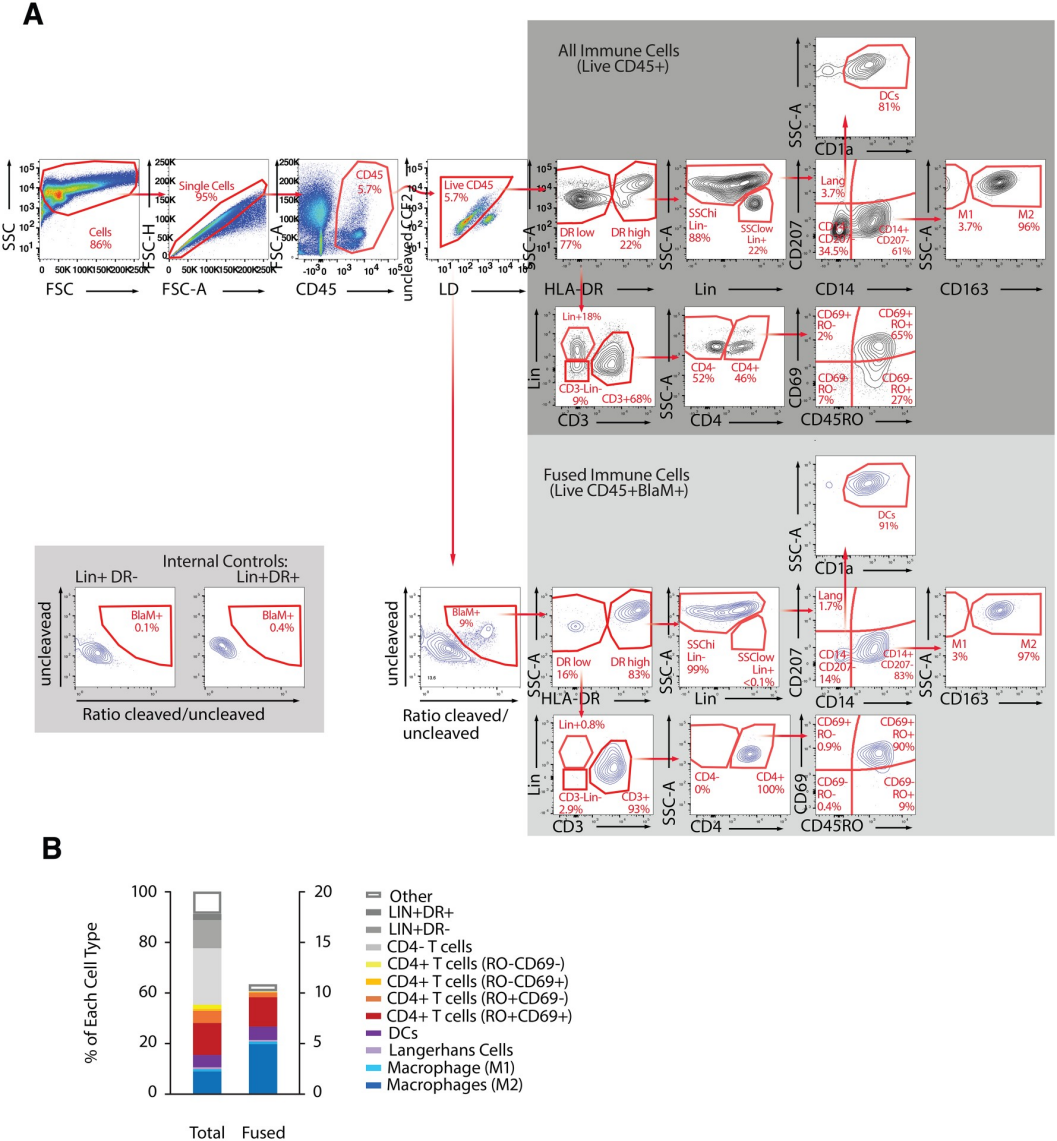
Fusion assay combined with immunostaining to identify and quantify phenotypes of immune cells from endometrium that support HIV-1 fusion. (A) Gating strategy: The first 4 multicolor FACS plots allow the identification of the “Live CD45^+^”, which represent the total number of immune present in the endometrial sample. From the “Live CD45^+^” gate, the “BlaM^+^ cells” can be identified and represent the immune cells that supported NL-109F4 viral fusion. The FACS plots on the gray background show the gating strategy to identify 11 cellular subsets among the “all immune cells” (dark gray) and among “fused immune cells” (light gray). Note that LIN^+^DR^-^ or LIN^+^DR^+^ can be used as internal control for gating the fused cells (bottom left inset). (B) For an endometrial biopsy sample from a single participant, paired bar graphs show the distribution of immune cell phenotypes (left) and prevalence of fusion susceptibility by phenotype (right). In this sample our immunophenotyping panel recognized the phenotype of more than 90% of the immune cells present in the biopsies, and overall fusion susceptibility was 13.6%.

BlaM-Vpr containing HIV-1 virions were produced by transfection of 293T cells with the indicated molecular clones as described [19] and the p24^Gag^ content measured with the FlaQ assay [20] to allow normalization of virus input to 500 ng p24^Gag^ in all assays. The fusion assay was conducted as previously described [19]. We had previously shown that triplicate infections were not needed and that the major source of variation was the donor of target cells [21]. Single cell suspensions were thawed and counted, and aliquots containing at least 5 x 10^4^ cells from cervical biopsies, 4 x 10^5^ cells from endometrial biopsies or 3 x 10^6^ cells from peripheral blood were infected for 1.5 h with BlaM-Vpr containing HIV-1 virions (500 ng p24^Gag^/ml). After infection the cells were washed and loaded with CCF2, the BlaM substrate, and incubated overnight at room temperature to allow cleavage by BlaM. Cells from FRT biopsies were stained with the FRT immunophenotyping panel composed of 11 antibodies:anti-CD3-BUV737 (BD Biosciences #564307), anti-CD4-BUV395 (BD Biosciences #563550), anti-CD14-BV650 (Biolegend # 301836), anti-HLA-DR-PerCP-Cy5.5 (Biolegend #307630), anti-CD45-BV605 (BD Biosciences #564047), anti-CD207 (Biolegend #352204), anti-CD45-RO-ECD (Beckman coulter #IM2712U), anti-CD163-PE-Cy7 (Biolegend #333614), anti-CD1a-A700 (Biolegend #300120), anti-CD69-APC7 (BD Biosciences #560737), anti-LIN [i.e. anti-CD56-APC (BD Biosciences #555518), anti-CD20-APC (BD Biosciences #559776) and anti-CD19-APC (BD Biosciences # 3404370)] and LIVE/DEAD Green (Invitrogen). We noticed some inconsistencies in fluorescence measurements for CD45RO-ECD marker for the cervical and endometrial samples. We elected not to use this marker and instead to phenotype the CD4+ T-cells using only CD69. Given that most of the cells in these FRT tissues are memory cells (Fig 1A), this omission had minimal impact on the phenotyping).

Cells from peripheral blood were stained with the immunophenotyping panel composed of 8 antibodies: anti-CD3-BUV737 (BD biosciences #564307), anti-CD4-BUV395 (BD biosciences #563550), anti-CD14-BV650 (Biolegend # 301836), anti-HLA-DR-PerCP5.5 (Biolegend #307630), anti-CD1c-PE (Affymetrix #12-0015-42), anti-CD303-PE-Cy7 (Biolegend #354214), anti-CD141-APC-Cy7 (Miltenyi # 130-098-217), anti-LIN (same as above) and LIVE/DEAD Green.

Cells from all sample types were fixed in phosphate-buffered saline containing 1.2% paraformaldehyde and acquired on a FACS Aria. Compensation beads were stained and acquired in parallel with the fusion assay. Compensation and gating were performed on Flow Jo Version X. The number of cells within each gate (immune cells present and number fused, by phenotype) was exported to an Excel spreadsheet for further analysis.

### Statistical methods

Within contraceptive groups we describe characteristics of the 58 healthy women participants who donated samples for this study (Table 1). For categorical characteristics we report counts and proportions and compare groups using Chi-square tests of independence with 3x(R-1) degrees of freedom (df), where R is the number of categories of the variable. For continuous characteristics we report median (and IQR or range) and Kruskal-Wallis tests with 3 df. We also report the number of samples available for analysis by type and group. Most experiments included multiple samples per woman, with replicates arising in two ways: 1) multiple single-cell suspensions created from a single endometrial sample, each infected with a distinct virus; and 2) distinct samples from three anatomic sites, all infected with the same virus.

**Table 1:** Characteristics of participants who contributed samples to the study.

	Control	COCs	Copper IUD	LNG-IUD	P-value
	n = 17	n = 14	n = 16	n = 11	
**Age (years)**					0.004 ^a^
Median (Min,Max)	32 (21,46)	23.5 (21,33)	25.5 (19,33)	26 (20,41)	
**Body Mass Index (kg/m**^**2**^**)**					0.023 ^a^
Median (Min,Max)	26 (20,55)	23 (19,35)	21(18,29)	24 (20,29)	
**Race**					0.001 ^b^
White	5(29%)	5 (36%)	11(69%)	8(73%)	
Black or African-American	9(53%)	1(7%)	0(0%)	1(9%)	
Asian	3(18%)	8(57%)	4(25%)	1(9%)	
Missing	0(0%)	0(0%)	1(6%)	1(9%)	
**Ethnicity**					0.11 ^b^
Hispanic or Latina	5(29%)	0(0%)	5(31%)	4(36%)	
Non-Hispanic	12(71%)	14(100%)	11(69%)	7(64%)	
**Education completed**					0.52 ^b^
Some high school	2(12%)	0(0%)	1(6%)	1(9%)	
Some college	5(29%)	5(36%)	3(19%)	2(18%)	
College or above	10(59%)	9(64.3%)	12(75%)	8(73%)	
**Current smoker**					0.071 ^b^
No	13(76.5%)	14(100%)	15(94%)	9(82%)	
Yes	4(23.5%)	0(0%)	0(0%)	2(18%)	
Missing	0(0%)	0(0%)	1(6%)	0(0%)	
**Parity**					0.13 ^b^
0	12 (71%)	14 (100%)	16 (100%)	9 (82%)	
1	1 (6%)	0(0%)	0(0%)	0(0%)	
2	4 (24%)	0(0%)	0(0%)	2 (18%)	
**Current contraceptive method use (months)**					0.29 ^a^
Median (Min,Max)	N/A	26.5 (9,42)	21.5 (6,45)	17 (7,36)	
**Sexual activity (vaginal sex, past 6 months)**					0.034 ^b^
None	5(29%)	2(14%)	0(0%)	0(0%)	
Some	12(71%)	12(86%)	16(100%)	11(100%)	
**Lifetime sex partners (#)**					0.29 ^a^
Median (Min,Max)	8 (1,50)	5 (1,23)	10 (2,30)	8 (1,20)	
**Samples analyzed (#)**					
Endometrial biopsy ^c^	14 [12]	12 [8]	15 [13]	10 [7]	
Cervical biopsy	15	11	16	8	
PBMC	14	15	13	11	

^a^ Kruskall Wallis test

^b^ Chi square test of association

^**c**^ S1 Fig: For virus ZM247Fv2, N = 51 samples infected. For viruses REJO and RHPA, N = 40 samples infected.

To convey methods for analyses of immune cells and their fusion to viruses, it is helpful to introduce some replicate-level notation. In biopsy and blood samples, staining by immunophenotyping panels allowed identification of *J* specific immune cell phenotypes; we grouped all other phenotypes as ‘other.’ We denote the *J* immune cell counts per replicate by *C*_*j*_, *j = 1*,*2*,*…*,*J*, which sum to the total, *C*_*total*_, and the *J* quantities of fused immune cells by *F*_*j*_, *j = 1*,*2*,*…*,*J*, which sum to the total, *F*_*total*_. We used these quantities in two ways. When describing individual replicates, we calculated the observed phenotypic distribution of immune cells by *C*_*j*_ */ C*_*total*_, *j = 1*,*2*,*…*, *J*, and of fused immune cells by *F*_*j*_ */ F*_*total*_, *j = 1*,*2*,*…*,*J*, and calculated the prevalence of HIV-1 fusion susceptibility (FS) per phenotype, *F*_*j*_ */ C*_*total*_, *j = 1*,*2*,*…*,*J*, and overall, *F*_*total*_ */ C*_*total*_. Therefore, all summaries of interest are proportions.

When evaluating groups of replicates, we used a generalized estimating equation (GEE) model with a logit link and binomial distribution to estimate a mean proportion from the components that form each ratio (e.g., *F*_*total*_ */ C*_*total*_) as a function of covariate(s) (e.g., phenotype X contraceptive group), and back-transform the logit-scale results to percentiles. All estimates of 95% confidence intervals (CI) are based on robust standard error (SE) and GEE score tests with 3 degrees of freedom (df) were used to assess statistically significant variation among contraceptive groups. To minimize inflation of estimates, participants were excluded from calculation of fused-cell distributions if *F*_*total*_ <6 (1 endometrial sample and 2 cervical samples). Statistical analyses were conducted using SAS version 9.4, with plots created using Excel.

**Fig 2:**
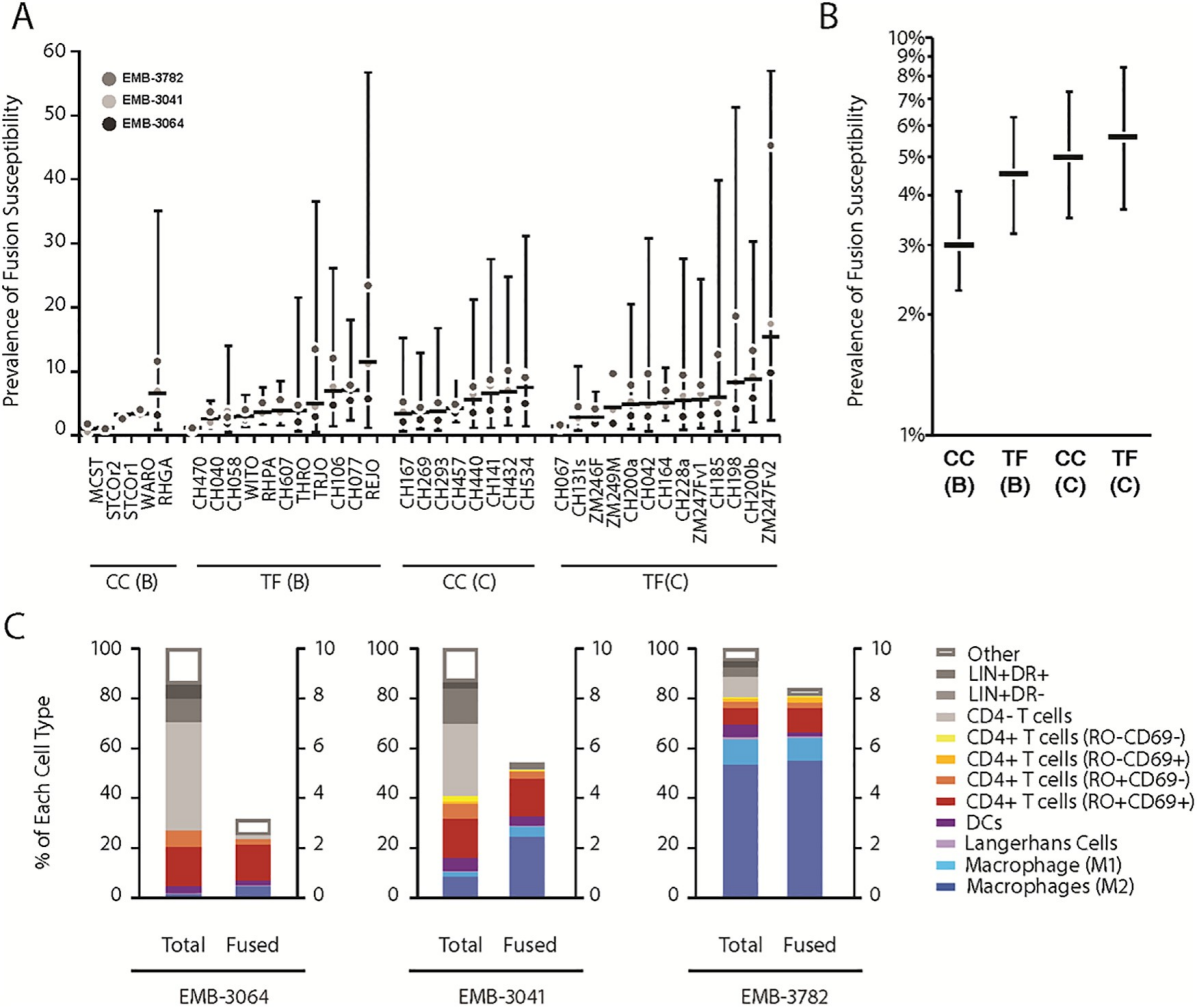
Fusion of 37 primary HIV-1 viruses to single cell suspensions from endometrial biopsies from 3 participants. Thirty-seven viruses containing BlaM-Vpr were obtained by co-transfection of 293T cells with infectious molecular clones and pcDNA4-3-BlaM-Vpr. Viral supernatants were concentrated and 500 ng p24^Gag^ was used to infect single cell suspensions generated from EMBs from three participants not using a contraceptive (EMB-3064, EMB-3041 and EMB-3782). The fusion assay was combined with immunostaining and gating as performed in Fig 1. (A) For each of 37 viruses, we display the sampling distribution of the prevalence of immune cells supporting fusion (i.e. % Live CD45^+^BlaM^+^ cells) based on n = 3 participants. Here, the horizontal bar represents the mean while the vertical bar indicates the 95% confidence interval (CI) based on Student’s t-distribution (2 df). (B) Mean (95% CI) fusion susceptibility of 37 viruses grouped by HIV-1 status at the time of viral isolation (founder [TF] vs. chronic [CC]) and subtype (B vs. C). (C) For each participant, distribution of immune cells and fusion susceptibility prevalence by phenotype, averaged over 37 viruses.

To illustrate replicate-level results generated by the fusion assay, for an endometrial biopsy sample and a blood sample, each from a single control participant not included in subsequent analyses, we present the observed distribution of immune cells (*C*_*j*_ */ C*_*total*_, *j = 1*,*2*,*…*,*12*) and FS prevalence by phenotype (*F*_*j*_ */ C*_*total*_, *j = 1*,*2*,*…*,*12*) using bar graphs. For Fig 2, using endometrial biopsy samples from n = 3 participants, immune cells and fused cells were quantified by phenotype (*C*_*j*_ and *F*_*j*_, *j = 1*, *2*,*…*,*12*, respectively) for each of 37 distinct viruses. In Fig 2A, for each virus, we estimated the mean (95% confidence interval) (CI) of the sampling distribution of fusion susceptibility prevalence. These CIs use the standard deviation (SD) rather than the standard error (SE), and the coefficient of Student’s t distribution with 2 degrees of freedom (df). Mean FS prevalence and robust SEs were generated via a generalized estimating equation (GEE) model of the ratio *F*_*total*_ */ C*_*total*_ as a function of virus, using a logit link and assuming a binomial distribution, accounting for correlated outcomes among viruses within participant. We then calculated the virus-specific standard deviation (SD) from the SE, calculated t-based confidence limits, and back-transformed the logit-scale mean (95% CI) to percentiles.

To describe variation in FS prevalence across viruses and participants, 37 replicates from endometrial samples donated by three participants were each infected with a distinct virus. We used control participants not using hormonal or IUD contraceptives to avoid measuring variation due to contraceptive effects; we used samples from three participants in order to get an appreciation for the variability between participant samples; the specific samples used were chosen at the beginning of the study based on high yield of tissue from the endometrial biopsies, since the assay required a large number of cells. For each virus, we estimated the sampling distribution of overall FS prevalence by: generating the mean and SD-based 95% CI via a GEE model of *F*_*total*_*/C*_*total*_ as a function of virus; calculating the SD from the robust SE and calculating SD-based confidence limits using the coefficient of Student’s t distribution with 2 df. To examine virulence by TF/CC status and B/C subtype, we estimated mean (95% CI) overall FS prevalence as a function of these strata, accounting for correlated outcomes among viruses within participant. (*C)* For each replicate, we calculated the observed immune cell distribution and FS prevalence by phenotype, then calculated arithmetic means across viruses for each participant.

To estimate immune cell abundance and susceptibility to HIV-1 infection, one replicate per available sample type was infected with virus ZM247Fv2 for each participant. All analyses were stratified by sample type (cervix, endometrium or blood). By phenotype, we summarized median counts and mean proportions of immune cells (*C*_*j*_ */ C*_*total*_), fused cells (*F*_*j*_ */ F*_*total*_), and FS prevalence (*F*_*j*_ */ C*_*total*_), where medians were generated via descriptive statistics and means were generated via GEE models of the respective ratio as a function of phenotype. In addition, we use boxplots to describe heterogeneity among participants in cell counts by phenotype.

To estimate the effect of contraceptive exposure on HIV-1 susceptibility, we reduced phenotype categories of interest to four, and grouped remaining phenotypes as ‘other.’ The first set used the samples described above, and stratified analysis by sample type. The second set used three replicates per participant from endometrial samples, each infected with one virus (ZM247Fv2, REJO, or RHPA) and stratified by virus. Using stratified GEE models, we estimated mean FS prevalence (*F*_*j*_ */ C*_*total*_) as a function of phenotype, contraceptive group, and their interaction; from the same models we estimated mean (95% CI) overall FS prevalence (*F*_*total*_ */ C*_*total*_) per contraceptive group. FS prevalence estimates are presented via bar graphs.

## Results

### Fusion assay adapted to cells from endometrial biopsies

The fusion assay allows quantification of HIV-1 entry into target cells [19] including those from the FRT [22] and relies on the transfer of BlaM-Vpr chimera from the HIV-1 virus to the target cells. This transfer can then be revealed by loading the cells with CCF2, the fluorescent substrate of BlaM. To profile the cells that supported fusion in endometrial and cervical TZ biopsies, we developed an immunostaining panel that allows identification of the various cell populations that support HIV-1 entry. The staining panel includes markers for CD4^+^ T-cells (CD3, CD4, CD45RO, CD69), macrophages (CD14, HLA-DR and CD163), DCs (CD1a) and Langerhans cells (CD207). Additionally, a set of lineage markers (CD56, CD19 and CD20) and a live/dead cell marker were included to facilitate the gating strategy.

#### Development of the assay using a laboratory-adapted HIV-1 strain

NL4-3 based provirus encodes the CCR5-tropic envelope found in transmitted founder virus 109F4 herein named NL-109F4 [21] (Fig 1). Single cells suspensions (4 x 10^5^ cells) from endometrial biopsies were infected for 1.5 h at 37°C with NL-109F4 containing BlaM-Vpr (500 ng of p24^Gag^). The fusion assay was conducted as previously described and combined with the immunostaining panel described above. Our gating strategy, presented in Fig 1A, was designed to (1) profile the phenotype of immune cells (CD45^+^) present in the biopsies (2) measure the total percentage of immune cells that supported HIV-1 fusion and (3) phenotype the immune cells that supported HIV-1 fusion. The fusion gate was defined using a FACS plot showing the ratio of fluorescence of cleaved to uncleaved substrate on the x axis and uncleaved substrate on the y axis. As expected, the fused cells were almost exclusively in cell populations known to express HIV-1 receptors (CD4^+^ T-cells, macrophages, dendritic cells [DCs] and Langerhans cells). As expected, LIN^+^ cells (mainly B and NK cells) did not support fusion and could be used as an internal control to establish the fusion gate (Fig 1A, bottom left inset). Our immunostaining panel could phenotype most of the immune cells that supported NL-104F4 fusion as evidenced by the very low number of fused cells not identified by our panel (classified as ‘other’) (Fig 1B). In the sample shown, ~13.6% of the total immune cells (i.e. CD45^+^ live cells) supported fusion of NL-109F4; we henceforth refer to the ratio of fused cells to total immune cells as the fusion susceptibility (FS).

#### Investigating fusion using a panel of 37 primary HIV-1 viral isolates

Given the high degree of diversity among HIV-1 isolates, we next investigated fusion mediated by a set of 37 circulating HIV-1 isolates that were previously cloned and characterized [23–26]. This set includes Subtype B and C HIV-1 species that were found either in the chronic phase of HIV-1 infection (CC) or were transmitted founder viruses (TF) found early in the course of infection. More details on these molecular clones are given in Table 2. BlaM-Vpr containing virions corresponding to these isolates were produced by transfection of 293T as described in Methods. Endometrial biopsies from 3 healthy women participants not on hormones or IUDs were used as the source of target cells for the set of 37 viruses (Fig 2). The estimated sampling distributions of FS varied within and between viruses (Fig 2A). For example, virus RHPA, REJO, and ZM247Fv2 had mean (95% CI) FS of 3.6% (range 1.6–7.5), 11.3% (range 1.2–57), and 15.3% (range 2.4–57), respectively.

**Table 2:** Characteristics of HIV-1 viral clones used in these experiments.

IMC	Subtype	Infection status^a^	Sex	Risk factor^b^	Country of origin	Reference
ZM249M	C	TF	M	HSX	Zambia	[23]
CH042	C	TF	M	HSX	South Africa	[24]
CH200a	C	TF	M	HSX	Malawi	[25]
CH106	B	TF	M	MSM	USA	[24]
CH607	B	TF	M	MSM	USA	[24]
**ZM247Fv2**	**C**	**TF**	**F**	**HSX**	**Zambia**	[26]
CH198	C	TF	M	HSX	South Africa	[24]
TRJO	B	TF	M	MSM	USA	[24]
CH131s	C	TF	M	HSX/MSM	Malawi	[25]
CH185	C	TF	F	HSX	South Africa	[24]
CH141	C	CC	F	HSX	Malawi	[25]
CH200b	C	TF	M	HSX	Malawi	[25]
CH293	C	CC	F	HSX	Malawi	[25]
CH534	C	CC	F	HSX	South Africa	[25]
CH164	C	TF	M	HSX/MSM	South Africa	[24]
**REJO**	**B**	**TF**	**M**	**HSX**	**USA**	[24]
CH067	C	TF	F	HSX	South Africa	[24]
CH457	C	CC	F	HSX	Tanzania	[25]
ZM247Fv1	C	TF	F	HSX	Zambia	[26]
CH040	B	TF	M	MSM	USA	[24]
CH432	C	CC	M	HSX	Malawi	[25]
**RHPA**	**B**	**TF**	**F**	**HSX**	**USA**	[24]
CH440	C	CC	F	HSX	Malawi	[25]
CH077	B	TF	M	MSM	USA	[24]
STCOr1	B	CC	M	MSM	USA	[25]
ZM246F	C	TF	F	HSX	Zambia	[25]
WARO	B	CC	F	HSX	USA	[25]
CH269	C	CC	F	HSX	Malawi	[25]
CH167	C	CC	F	HSX	Malawi	[25]
CH228a	C	TF	M	HSX	Malawi	[25]
MCST	B	CC	M	MSM	USA	[24]
CH470	B	TF	M	MSM	USA	[24]
WITO	B	TF	M	HSX	USA	[24]
THRO	B	TF	M	MSM	USA	[24]
RHGA	B	CC	M	MSM	USA	[25]
CH058	B	TF	M	MSM	USA	[24]
STCOr2	B	CC	M	MSM	USA	[25]

Rows in bold text are the viruses used for comparison of fusion in the different contraceptive groups.

^**a**^ TF: transmitted founder; CC: chronic control

^**b**^ MSM: men who have sex with men; HSX: heterosexual exposure.

Although the FS was lowest in EMB-3064 and highest in EMB-3782 for most viruses, the values fell within the respective 95% confidence intervals of the sample mean (Fig 2A). When viruses were classified according to their subtype B or C and whether they were identified in early (TF) or in the chronic phase of infection (CC), there were no major differences in mean FS prevalence by virus subtypes or phases of infection. Fig 2B shows the mean and 95% confidence interval for each stratum. While the TF viruses and subtype C viruses appeared to be more fusogenic, the high degree of variability among viruses in a sample of 3 participants precluded reliable comparison via a statistical test.

When we investigated the prevalence of FS across the major cellular subsets (Fig 2C), we found that M2 macrophages (CD14^+^CD163^+^) and activated CD4^+^ T-cells (RO^+^CD69^+^) accounted for the majority of the fused cells in cervix and endometrium. DCs and Langerhans cells accounted for small portions of fused cells likely because of their low abundance in the samples. As expected, fusion to CD4^-^ T-cells, B cells (i.e., LIN^+^HLA-DR^+^ cells) and NK cells (LIN^+^ HLA-DR^-^ cells) was minimal.

### Fusion assay adapted to cells from peripheral blood

To profile the cells that supported fusion in peripheral blood mononuclear cells samples, we tailored the immunostaining panel to allow for phenotyping of immune cells known to be present in peripheral blood, which differ from those in the endometrium and cervix. The staining panel includes markers for CD4^+^ T-cells (CD3, CD4, CD45RO, CD69), monocytes (CD14, HLA-DR), plasmacytoid DC (pDC) (CD303), and myeloid DC (CD1c, D141); DC characterization was performed as previously recommended [27]. Additionally, a set of lineage markers (CD56, CD19 and CD20) and a live/dead cell marker (Invitrogen) were included to facilitate gating.

**Fig 3:**
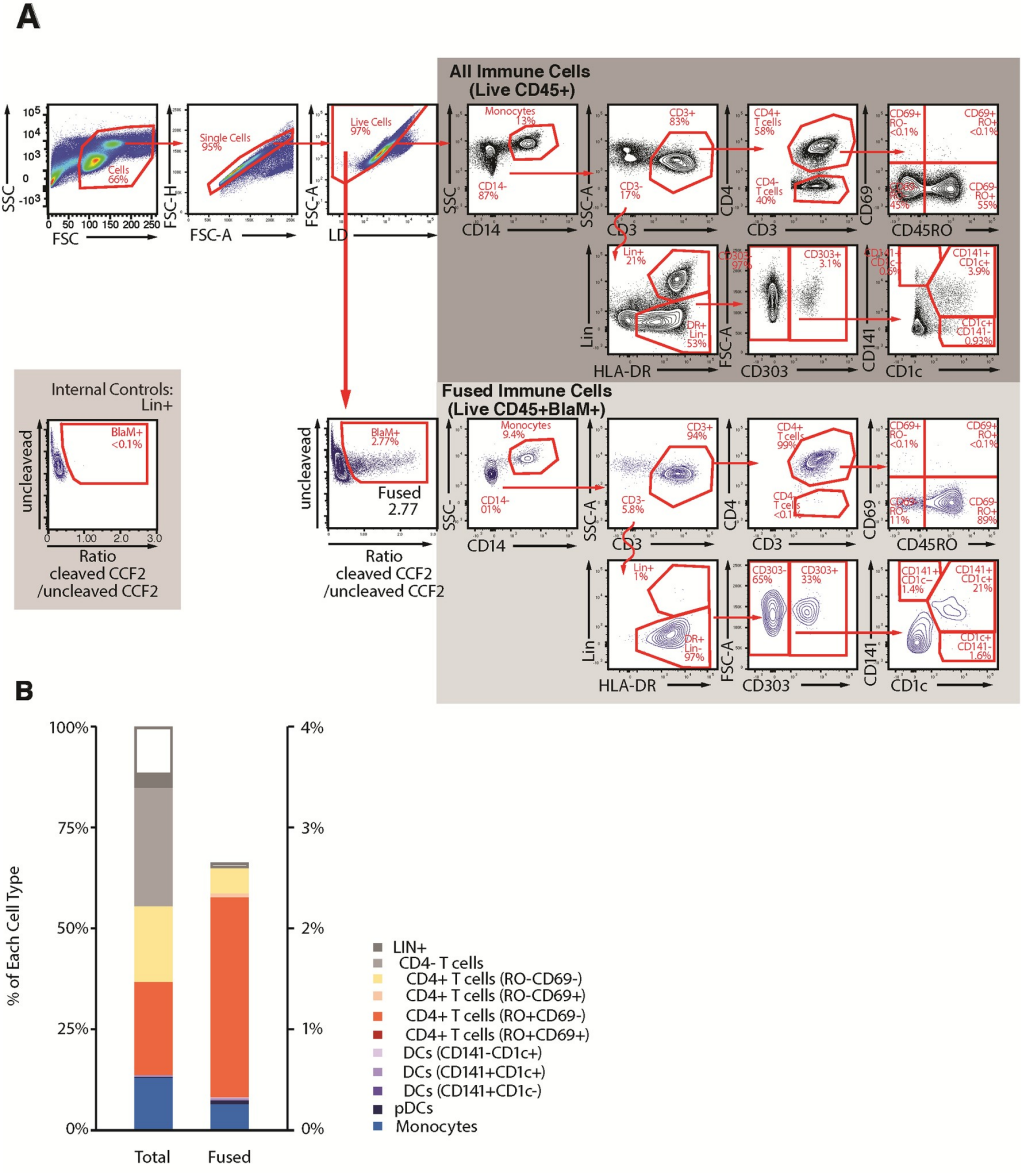
Fusion assay combined with immunostaining allows measurement of the percentage and phenotypes of immune cells that support HIV-1 fusion in cells from peripheral blood. (A) Gating strategy: The first 4 multicolor FACS plots allow the identification of the “Live cells”. From this gate, the “BlaM^+^ cells” can be defined and represent the immune cells that supported ZM247v2 viral fusion. The FACS plot on the gray background show the gating strategy to identify 11 cellular subsets among the “all immune cells” (dark gray) and among “fused immune cells” (light gray). Note that LIN^+^ can be used as internal control for gating the fused (bottom left inset). (B) For a blood sample from a single participant, paired bar graphs show the distribution of immune cell phenotypes (left) and prevalence of prevalence of fusion susceptibility by phenotype (right). In this sample our immunophenotyping panel recognized the phenotype of more than 85% of the immune cells present in blood, and overall prevalence of fusion susceptibility was 2.6%.

Given that peripheral blood cells are known to harbor relatively low numbers of cells susceptible to HIV-1 entry [21], the fusion assay was conducted using more cells than used for the endometrial and cervical samples (3 x 10^6^ cells per sample). These cells were infected for 1.5 h with 500 ng p24^Gag^ of ZM247v2 and the fusion assay was conducted as described above. Fig 3A illustrates the gating strategy employed to identify the major subsets of HIV-1 susceptible cells in the blood (CD4^+^ T-cells, monocytes, pDC, and myeloid DC). As for the endometrial and cervical cell analysis, this gating strategy was also designed to (1) profile the phenotype of immune cells (2) measure the total percentage of immune cells that supported HIV-1 fusion; and (3) phenotype the immune cells that supported HIV-1 fusion. As expected, LIN^+^ cells (mostly B and NK cells) did not support fusion and could be used as an internal control to set the fusion gate (Fig 3A, bottom left plot). PBMCs are known to harbor few activated CD4^+^ T memory cells (CD45RO^+^CD69^+^) in healthy individuals, and the CD69- memory CD4^+^ T-cells accounted for the majority of fusion-susceptible cells in the blood (Fig 3B). Monocytes and pDC were also susceptible to entry by ZM247v2.

### Characterization of participants in the four contraceptive groups

To investigate the effects of contraceptive use on susceptibility to HIV-1 infection, we studied samples from women using LNG-containing IUDs, LNG-containing COCs, copper IUDs, and women not on hormonal or intrauterine contraceptives (control group) as described in Methods. Table 1 shows the numbers and demographic characteristics of participants in each group who contributed samples to this study. Although we planned to balance the sample size by group, for logistical reasons we closed accrual after 11 to 17 eligible participants per group were enrolled. The number of participants available for specific analyses depended on sample type (endometrium n = 51, cervix n = 50, and blood n = 53) and the virus studied (Table 1 footnote).

Controls differed from the contraceptive-exposed groups with respect to age, body mass index, race, and frequency of sexual intercourse (Table 1). With regards to biologic variables, the median progesterone levels at the time of sample collection was 9.5 ng/ml (range 0.5–18.6) in the control group and 10.9 ng/ml (range 0.8–27.8) in the copper IUD users, demonstrating that the majority of women in those groups had ovulated. The median progesterone level in the LNG-IUD group was much lower at 1.8 ng/ml (range 0.5–14.5) and is consistent with our finding that the majority of women in the LNG-IUD group did not detect an LH surge over a 2-month testing period. The median progesterone level in the COC group was 0.5 ng/ml (range 0.5–1.0), consistent with suppression of ovulation by COC use. We verified that the women on LNG-containing contraceptives had measurable LNG levels in their blood; the median LNG level for OC users was 4.23 ng/ml (range 0.7–6.8) and for LNG-IUD users was 0.18 (range 0.1–0.3). The median vaginal pH was 4.4 in all groups, with a range of 3.6–6.1. One participant in the control group had a positive test for PSA, indicating recent sexual intercourse.

### Immune cell phenotypes and HIV-1 fusion susceptibility with ZM247Fv2 to cells from 3 anatomic locations

Samples collected over a 2-year period were frozen in liquid nitrogen. To limit experimental variability, the fusion assay was conducted at a single time for all samples from the same anatomic site. Fusion was tested with ZM247Fv2, the highly fusogenic TF subtype C virus, for all sample sites.

#### Endometrium and cervix samples

**Fig 4:**
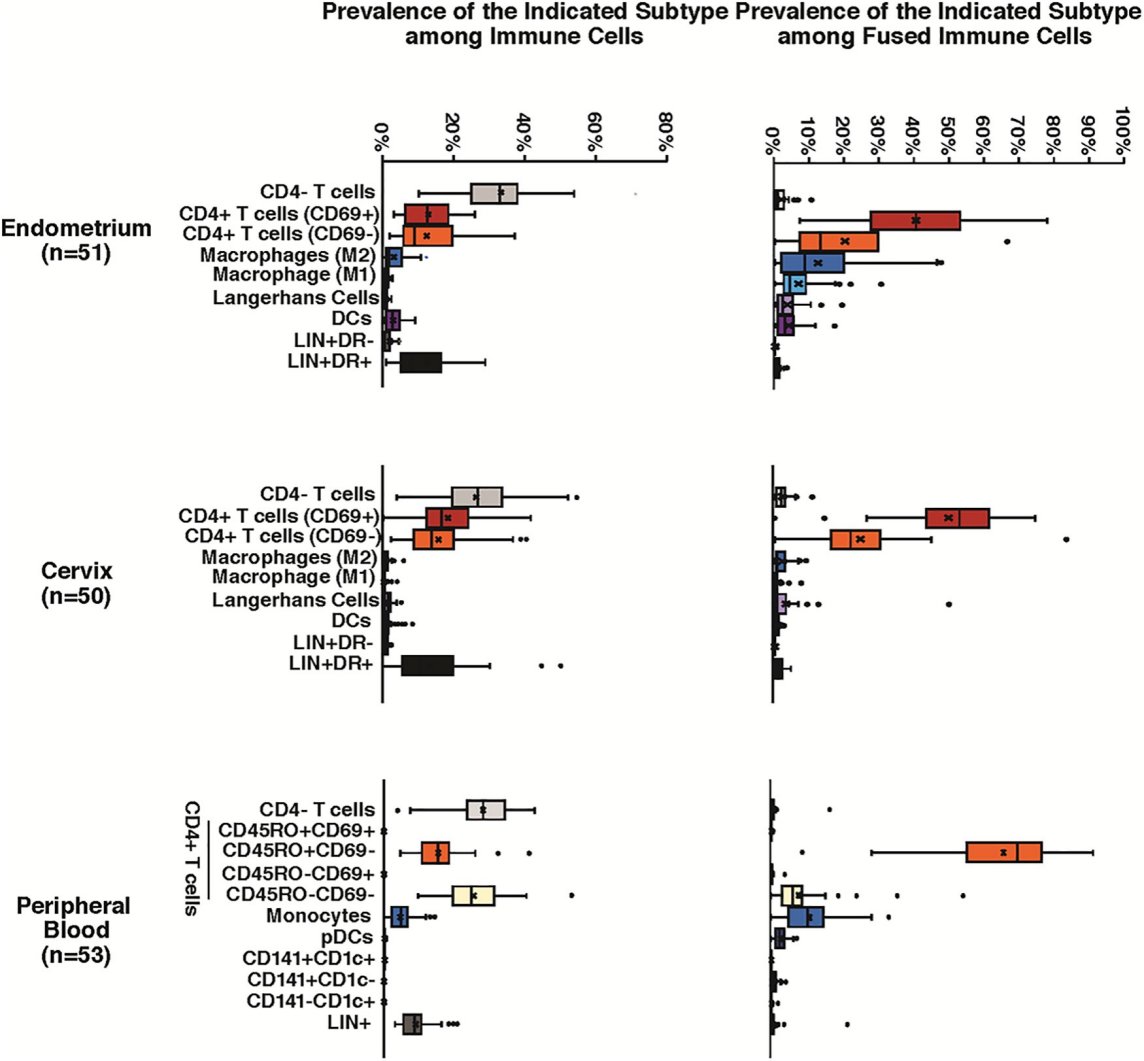
Distribution of cellular subsets in total and fused immune cells from 3 anatomic locations. Single-cell suspensions from endometrium (n = 51), cervix (n = 50), and peripheral blood (n = 53) were infected with ZM247v2 HIV-1 virions containing BlaM-Vpr. All infections were performed simultaneously for a given anatomical site. After 1.5 h of infection the fusion assay was conducted. Box plots are used to describe the distributions of immune cells (left) and fused immune cells (right) for 11 cellular subsets per sample location. The line through the box represents the median, and the x represents the mean; whiskers on the box plots represent the 255h and 75^th^ percentiles; outliers are indicated with black circles.

We used the gating strategy described in Fig 1 to assess the distributions of immune cells across all participants for endometrium (n = 51) and cervix (n = 50). We found higher proportions of macrophages and dendritic cells in the endometrium and more CD4^+^ T-cells and Langerhans cells in the cervix (Fig 4 and Table 3), but collectively, the sum of these four HIV-1 target cells amongst all immune cells was similar for the 2 sites (33% for cervix and 40% for endometrium). Fig 4 reveals substantial inter-individual variability in the proportion of each cell type, both among all immune cells and among cells that fused to HIV-1 virus ZM247Fv2.

**Table 3:** Estimates of distributions of immune cells, fused cells and fusion susceptibility by immune cell phenotype in ZM247Fv2-infected endometrial and cervical samples. (See also Fig 4 for variation among participants in distributions of immune cells and fused cells.).

Immune cell phenotype	Endometrium (n = 51)			Cervix (n = 50)		
	Immune cells^a^ Median^c^ # (Mean %)	Fused cells^b^ Median^c^ # (Mean %)	Fusion susceptibility^a^ Mean %	Immune cells^a^ Median^c^ # (Mean %)	Fused cells^b^, Median^c^ # (Mean %)	Fusion susceptibility^a^ Mean %
Total	2156 (100%)	218 (100%)	9.11%	2578 (100%)	235 (100%)	8.70%
Macrophages	74 (3.66%)	27 (18.2%)	1.66%	31 (1.19%)	5 (2.23%)	0.19%
M2	40 (2.09%)	16 (11.8%)	1.07%	18 (0.79%)	3 (1.72%)	0.15%
M1	29 (1.66%)	9 (6.26%)	0.57%	7 (0.37%)	1 (0.49%)	0.04%
Langerhans	16 (0.76%)	4 (3.06%)	0.28%	31 (1.38%)	3 (1.61%)	0.14%
Dendritic cells	42 (2.30%)	7 (3.60%)	0.33%	25 (0.97%)	2 (0.65%)	0.06%
CD4^-^ T-cells	796 (30.2%)	3 (1.49%)	0.14%	493 (24.6%)	3 (2.69%)	0.23%
CD4^+^ T-cells	480 (26.4%)	127 (63.7%)	5.80%	812 (36.2%)	188 (80.9%)	7.04%
RO^+^ CD69^+^	252 (12.1%)	79 (43.1%)	3.93%	467 (19.2%)	108 (53.9%)	4.69%
RO^+^ CD69^-^	192 (13.7%)	32 (19.7%)	1.80%	331 (16.1%)	47 (24.8%)	2.16%
LIN^+^ DR^-^	19 (1.79%)	0 (0.0%)	0.00%	25 (1.29%)	0 (0.0%)	0.00%
LIN^+^ DR^+^	223 (15.9%)	1 (1.23%)	0.11%	271 (15.3%)	2 (1.58%)	0.14%
Other ^d^	403 (19.0%)	19 (8.71%)	0.79%	411 (17.2%)	26 (10.3%)	0.90%

^a^ Denominator is total number of immune cells per sample type; all phenotypes have *C*_*total*_ *> 200*.

^b^ Denominator is total number of fused immune cells per sample type. Column excludes three samples with *F*_*total*_ *<6* (one endometrium, 2 cervix).

^c^ Counts (#) are medians across n = 51 (endometrium) and n = 50 (cervix) participants. For medians, sum of components can differ from total. Percents (%) are means estimated from generalized linear models (binomial distribution with logit link).

^d^ Other = All–(Macrophages + Langerhans + Dendritic cells + CD4^-^ + CD4^+^ + LIN^+^)

Regarding the types of cells that supported fusion of ZM247Fv2, approximately 60% and 80% of fused cells were CD4^+^ T-cells for endometrium and cervix, respectively, with most of these cells expressing CD69 (Fig 4 and Table 3). The CD4^+^CD69^-^ T-cells accounted for a lower proportion of fused immune cells than the CD4^+^CD69^+^ cells, despite being present at roughly similar mean quantities. In the endometrial samples, macrophages were the next most common cell supporting fusion, at 18.2% of fused cells with the majority being M2 macrophages (11.8% M2 and 6.3% M1); in cervix the proportion of macrophages amongst fused cells was much lower at 2.2%.

The overall susceptibility of immune cells to ZM247Fv2 fusion was approximately 9% in both endometrium and cervix (Table 3), with CD4^+^ T-cells accounting for the majority of susceptible cells (5.80 and 7.04% respectively). In endometrium, M2 macrophages were the next most susceptible cell type (1.07%). In cervix, given that the numbers of macrophages were much lower, the measurements were therefore less reliable, and prevalence of FS was <1% for macrophages and for all other immune cell phenotypes.

#### Peripheral blood cells

We used the gating strategy described in Fig 3 to determine the distributions of immune cells in peripheral blood across all participants (n = 53). Of note, direct comparisons to the FRT sites could not be made due to differences in the phenotype panels. As seen in Fig 4, there was substantial variability among individuals in the quantity of each phenotype, similar to what was seen in FRT samples. Memory CD4^+^ T-cells accounted for 67% of the cells that supported fusion of ZM247v2 (Table 4). Monocytes, despite their relatively low abundance in blood (5.5%), accounted for 12.2% of fused cells; pDCs represented only 0.2% of immune cells in blood but 2.3% of fused immune cells. These results indicate a high HIV-1 susceptibility of monocytes and pDCs to HIV-1 entry. CD4^-^ T-cells and LIN^+^ cells were not represented among the fused cells, as expected, despite their high representation in the samples, demonstrating the specificity of the assay.

**Table 4:** Estimates of distributions of immune cells, fused cells and fusion susceptibility by immune cell phenotype in ZM247Fv2-infected peripheral blood cells.

Immune cell phenotype	Immune cells^a^ Median #^c^ (Mean %)	Fused cells^b^ Median #^c^ (Mean %)	Fusion susceptibility^a^ Mean %
Total	524,000 (100%)	5,771 (100%)	1.28%
Monocytes	27,441 (5.5%)	724 (12.2%)	0.16%
LIN^-^	30,813 (7.0%)	517 (8.2%)	0.10%
CD14^-^ CD3^-^ DR^+^ LIN^-^CD303^-^	29,986 (6.8%)	322 (5.7%)	0.074%
Myeloid DCs: CD141^-^ CD1c^+^	252 (0.056%)	6 (0.11%)	0.001%
Myeloid DCs: CD141^+^ CD1c^-^	101 (0.021%)	4 (0.086%)	0.001%
Myeloid DCs: CD141^+^ CD1c^+^	1,043 (0.22%)	51 (1.02%)	0.013%
Other antigen-presenting cells: CD141^-^CD1c^-^	28,380 (6.5%)	257 (4.8%)	0.063%
Plasmacytoid DCs	894 (0.2%)	142 (2.3%)	0.030%
CD4^-^ T-cells	149,300 (29.0%)	28 (0.58%)	0.007%
CD4^+^ T-cells	196,756 (39.3%)	4,212 (76.3%)	0.98%
CD4^+^ T-cells (RO^-^ 69^-^)	115,909 (24.6%)	308 (8.4%)	0.11%
CD4^+^ T-cells (RO^-^ 69^+^)	135 (0.030%)	12 (0.21%)	0.003%
CD4^+^ T-cells (RO^+^ 69^-^)	71,936 (14.8%)	3,887 (66.5%)	0.87%
CD4^+^ T-cells (RO^+^ 69^+^)	109 (0.022%)	9 (0.15%)	0.002%
Other cell types	57,370 (10.0%)	45 (1.28%)	0.033%

^a^ Denominator is the total number of immune cells.

^b^ Denominator is the total number of fused cells.

^c^ Counts (#) are medians across 53 participants. For medians, sum of components can differ from total.

Percents (%) are means estimated from generalized linear models (binomial distribution with logit link).

Overall, FS was higher in endometrial and cervical samples than in peripheral blood (9.11% versus 8.70% versus 1.29% respectively, Tables 3 and 4). FS was highest in CD4^+^ T-cells in all 3 anatomic sites (5.9, 7.0 and 0.98% respectively). None of the subsets of blood cells had >1% FS, demonstrating the overall low susceptibility of blood cells to HIV-1 entry.

### The effect of contraceptives on HIV-1 entry into target cells from endometrium, cervix and blood

We investigated whether the type of contraceptive used by the women participants influenced FS overall or by cell phenotype in endometrial, cervical, and blood samples. To limit the influence of subsets represented at low levels, we grouped activated and non-activated CD4^+^ T-cells under the category of CD4^+^ T-cells, and M1 and M2 macrophages under the category of macrophages. Likewise, all the CD4^+^ T subtypes from peripheral blood were grouped under the larger category of CD4^+^ T-cells.

#### Fusion susceptibility with ZM247Fv

**Fig 5:**
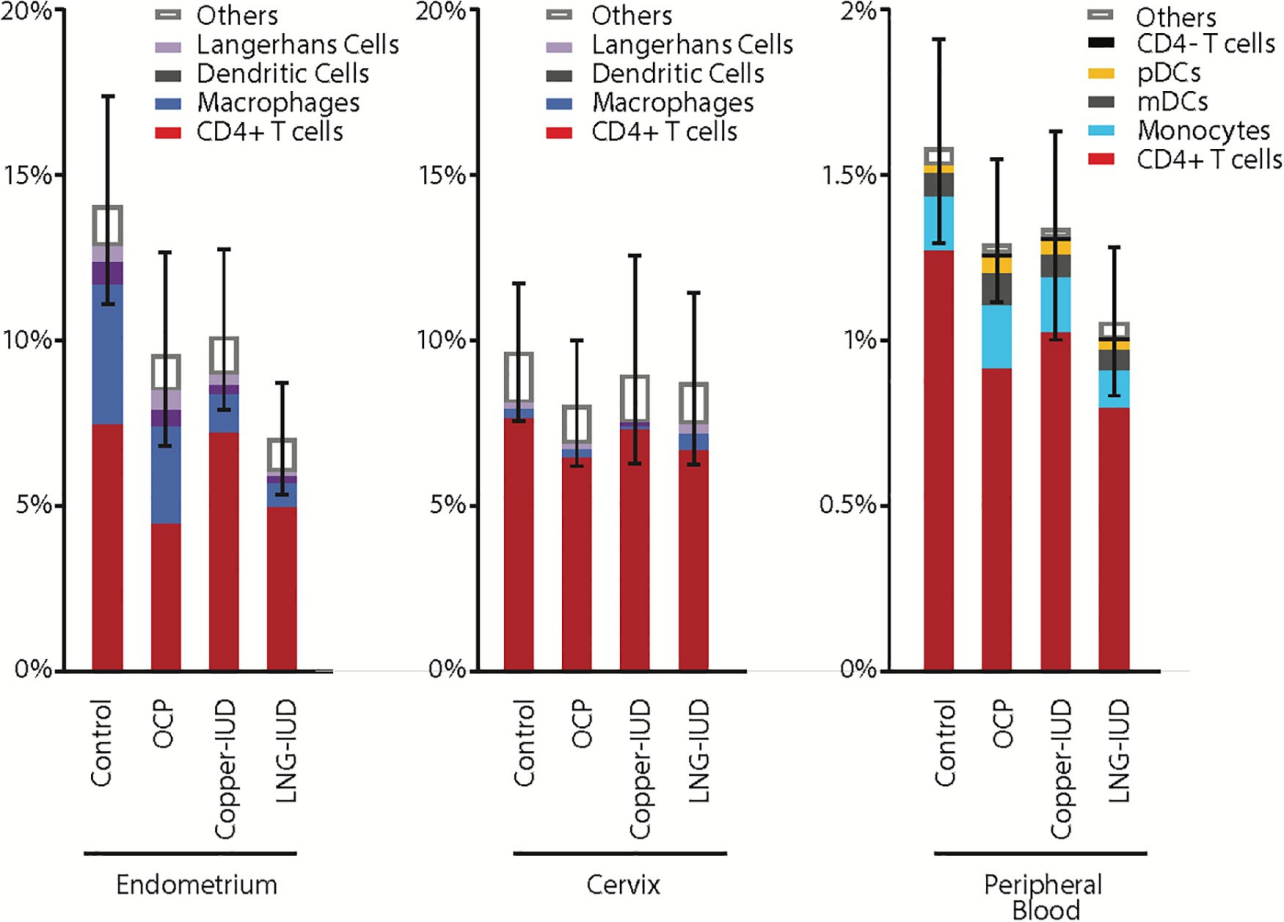
Effect of contraceptives on HIV-1 acquisition as measured by prevalence of fusion susceptibility of cells from 3 anatomic locations to HIV-1 fusion *ex vivo*. Bar graphs depict the mean prevalence of immune cells supporting ZM247v2 fusion (overall height of the bar, with confidence limits) by donor contraception group. Contributions of four major cellular subsets to the overall prevalence are identified by color clade. Note that the vertical axis is 10-fold greater for biopsy samples than blood samples. OCP is combined oral contraceptive.

For all sample types, viral fusion was higher among contraceptive non-users than contraceptive users (Fig 5); the differences were significant for the endometrial samples (Table 5), discussed in detail below. Fusion varied among immune cell phenotypes (p<0.001), with CD4^+^ T-cells accounting for the majority of cells that supported ZM247Fv2 fusion. The proportions of “other” cells, which are the immune cells that fell into the fusion gate but for which the gating and immunophenotyping did not lead to a clear classification, were similarly low across the groups, indicating that the immunostaining panel allowed phenotyping of the majority of fused immune cells in all of the participant groups.

**Table 5:** Generalized estimating equation model-based estimates of mean (95% CI) prevalence of fusion susceptibility of immune cells from ZM247Fv2-infected samples, modeled separately for each sample type, as functions of cell phenotypes shown and contraceptive exposure group (H = hormone +/-; IUD = intrauterine device +/-).

Immune cell phenotype	Control H^-^; IUD^-^	COC H^+^; IUD^-^	Cu-IUD H^-^; IUD^+^	LNG-IUD H^+^; IUD^+^	Overall^a^	P-value^b^
**Endometrium**	**(n = 14)**	**(n = 12)**	**(n = 15)**	**(n = 10)**	**(n = 51)**	
All immune cells	14% (11–17%)	9.4% (6.9–13%)	10% (7.9–13%)	6.9% (5.4–8.8%)	9.8% (8.6–11%)	0.026
CD4^+^ T-cells	7.42%	4.48%	7.11%	4.98%	5.87%	
Macrophages^c^	4.20%	2.85%	1.22%	0.70%	1.80%	
Dendritic cells	0.72%	0.54%	0.29%	0.14%	0.36%	
Langerhans cells	0.47%	0.59%	0.28%	0.07%	0.27%	
Other	1.13%	0.94%	1.11%	1.02%	1.05%	
**Cervix**	**(n = 15)**	**(n = 11)**	**(n = 16)**	**(n = 8)**	**(n = 50)**	
All immune cells	9.6% (7.7–12%)	8.0% (6.3–10%)	9.1% (5.7–12%)	8.6% (6.4–11%)	8.8% (7.5–10%)	0.75
CD4^+^ T-cells	7.72%	6.39%	6.95%	6.63%	7.1%	
Macrophages^c^	0.19%	0.23%	0.10%	0.44%	0.21%	
Dendritic cells	0.06%	0.05%	0.04%	0.10%	0.06%	
Langerhans cells	0.19%	0.12%	0.08%	0.20%	0.14%	
Other	1.47%	1.14%	1.29%	1.24%	1.28%	
**PBMC**	**(n = 14)**	**(n = 13)**	**(n = 15)**	**(n = 11)**	**(n = 53)**	
All immune cells	1.56% (1.3–1.9%)	1.28% (1.1–1.5%)	1.32% (1.0–1.8%)	1.04% (0.8–1.3%)	1.29% (1.1–1.4%)	0.14
CD4^+^ T-cells	1.27%	0.91%	1.02%	0.78%	0.99%	
Monocytes	0.16%	0.19%	0.16%	0.12%	0.16%	
CD14^-^ CD3^-^ DR^+^ LIN^-^ CD303^-^	0.069%	0.098%	0.073%	0.060%	0.075%	
Plasmacytoid dendritic cells	0.025%	0.046%	0.035%	0.020%	0.032%	
CD4^-^ T-cells	0.006%	0.006%	0.008%	0.012%	0.008%	
Other	0.034%	0.034%	0.022%	0.045%	0.034%	

^a^ In this table, overall estimates of fusion susceptibility are adjusted for contraceptive group.

^b^ P-values comparing exposure groups, adjusted for cell phenotypes shown, are based on 3-df score statistics for Type 3 generalized estimating equation analysis using robust SE.

^c^ Macrophages = M1+M2 macrophages combined

In endometrial samples, FS varied significantly among contraceptive groups both overall (P = 0.026; Table 5) and within the four major cell phenotypes (CD4^+^ T cells, macrophages, dendritic cells and Langerhans cells). In particular, significantly higher FS in the non-IUD groups as compared to the IUD groups (Control and COC versus IUDs, P = 0.010) was associated with above-average fusion of three subsets of cells (macrophages, Langerhans and dendritic cells; P = 0.007), whereas significantly higher FS in non-hormone groups as compared to the hormone groups (Control and Copper IUD versus COC and LNG-IUD p = 0.007) was not associated with a particular cell type.

In cervical and blood samples, the percent of immune cells supporting fusion varied little among contraceptive groups (overall P = 0.70 and 0.14, respectively; Fig 5 and Table 5). In cervical samples, FS of macrophages varied by contraceptive group (P = 0.032), being highest in the LNG-IUD group; however, very few cells were available for analysis (Table 3). In blood samples, FS of pDC varied by contraceptive group (P = 0.01); although the cell count was sufficient for analysis (Table 4), no clear association with either hormone or IUD exposure emerged (Table 5).

#### Fusion susceptibility with additional HIV-1 isolates in endometrial samples

All results above apply to ZM247Fv2 entry. We also compared FS by contraceptive group in those endometrial samples that had sufficient cell numbers using 2 additional HIV-1 isolates: REJO, a subtype B TF virus found in a newly infected male (n = 40 samples), and RHPA, a subtype B TF virus found in a newly infected woman (n = 40 samples) (S1 Fig). This analysis was limited to endometrial samples as insufficient cell numbers were available from the cervix. ZM247Fv2 and RHPA were the most and least fusogenic, respectively, consistent with the patterns seen from the three participants shown in Fig 2. In each case, CD4^+^ T-cells explained most of the FS and the proportions explained by “other” cells were low.

For viruses ZM247Fv2, REJO, and RHPA, FS was highest in each control group (14%, 10%, and 3.5%, respectively) and lowest in the LNG-IUD group (6.9%, 5.7%, and 1.3%), yielding relative Control:LNG-IUD ratios of 2.0, 1.8, and 2.7. In turn, the 3-df p-values testing for variation in FS were P = 0.026, P = 0.12, and P = 0.024, respectively. As shown in S1 Fig, FS of CD4^+^ T-cells was substantially higher in the groups not exposed to LNG (Control and copper IUD). FS of macrophages was higher in groups not exposed to a device and decreased across groups in the order displayed (Control > COC > Cu-IUD > LNG-IUD).

## Discussion

Our study was designed to assess the effects of hormonal and intrauterine contraceptives, separately and combined, on susceptibility to HIV-1 fusion. The analysis of 154 samples from 58 participants and 3 anatomical sites (endometrium, cervical transformation zone and peripheral blood) demonstrated that CD4^+^ T cells are the most susceptible cell type in all 3 anatomic locations, and that fusion was significantly higher in cells from cervix and endometrium than in blood. Our results confirm other reports that endometrial macrophages are highly susceptible to HIV-1 infection [28]. We also found that cells from women using LNG-containing COCs, LNG-IUDs and copper IUDs are not associated with increased susceptibility to HIV-1 fusion. In fact, the control group had the highest susceptibility to *ex vivo* fusion in samples from both the FRT and peripheral blood, a finding that was statistically significant and consistent amongst 3 different viral isolates. These results contribute to the knowledge base about hormonal contraceptives and IUDs in light of recent concerns about possible adverse effects of hormonal contraceptives on HIV-1 susceptibility, and are consistent with recent results from a randomized trial in women that showed no difference in HIV risk amongst women using depo-medroxyprogesterone acetate, a copper IUD and an levonorgestrel implant [10].

Our results show that different HIV-1 viruses have high variation in fusogenicity to primary cells from the cervix and endometrium. While TF subtype C viruses seemed to enter targets cells more efficiently than the other subtypes (Fig 2B), the sample size was too small to draw definitive conclusions regarding the effects of HIV-1 subtype or the phase of infection at the time when the virus was cloned (transmitted/founder virus and chronic infection) on HIV-1 fusogenicity to endometrial cells. Our data confirms the findings of a previous report that indicated that envelopes of transmitted/founder or control/reference viruses have similar infection patterns of CD4^+^ T-cells in human cervical tissue *ex vivo* [24].

We found a wide range of distribution of immune cell types susceptible to HIV-1 fusion, and variations in the extent of fusion depending on the participant. This range is indicated by the wide confidence intervals surrounding the medians in Figs 2 and 4. Fig 2C directly compares the distributions of immune cells and of fused cells in endometrial samples from 3 women and shows that the sample from participant EMB-3782 had a much higher proportion of macrophages and a correspondingly higher proportion of fusion events. Seminal fluid introduced into the vagina from coitus induces an influx of macrophages and dendritic cells into the cervix [29], and presumably a similar event could occur in the endometrium. However, our participants were instructed to use condoms throughout the study period and to refrain from intercourse for 72 hours prior to sample collection. We also collected a vaginal swab for prostate specific antigen detection at the time of sample collection as a marker for recent intercourse, which was negative for all of the samples in Fig 2, indicating that the high proportion of macrophages in sample EMB-3782 is unlikely to result from recent intercourse. To reduce the impact of infections on study samples, we screened women for sexually transmitted infections at study entry, and performed vaginal pH and when indicated, a wet mount, to exclude women with bacterial vaginosis at the time of sample collection. We conclude that the variation in immune cellular composition that we observed in endometrial and cervical samples may be attributable to the natural variation between women.

Our study has several strengths. This is the first direct comparison of HIV-1 fusion events in samples from women on hormonal and non-hormonal IUDs and COCs. We restricted the type of oral contraceptives to those containing LNG in a 28-day pill pack. Our collection of samples synchronized to the luteal phase (for cycling women), or to the latter part of the pill pack (for women on COCs), is meant to reduce biological variability due to hormonal fluctuations across the menstrual cycle. We used clones of primary HIV-1 isolates from different times in the infectious life cycle and of different subtypes. The fusion assay is performed on target cells that have not been externally activated or cultured, and hence are more likely to reflect the status of cells within their local environment than assays that measure downstream events in the HIV-1 viral lifecycle. We used state-of-the-art statistical methods to look for contraceptive effects on fusion susceptibility. Our study also has limitations. The control group differed from the other groups in age and other demographic characteristics representative of women who choose different methods. The relatively small sample sizes precluded rigorous statistical analyses of some comparisons. We were unable to time sample collection to precise times in the menstrual cycle in women on LNG-IUD because the majority were anovulatory. Participants had used contraceptive methods for varying lengths of time. Finally, the fusion assay is a surrogate for HIV-1 infection and therefore these results may not accurately reflect susceptibility *in vivo*. For example, epithelial cells could respond to the local hormonal environment by producing cytokines which could in turn affect HIV replication. Likewise, the microbiota could be influenced by the hormonal context and subsequently influence HIV replication [30]. These factors were not directly addressed by the *ex vivo* fusion assay.

In summary, we found that the use of LNG-containing COCs, LNG-IUDs and the copper IUD were not associated with increased HIV-1 fusion susceptibility of immune cells from the endometrium, cervix or peripheral blood. These findings are reassuring given the high numbers of women worldwide using such devices, and in light of our previous work showing an increase in the numbers of activated CD4^+^ T-cells in endometrial samples from LNG-IUD users compared to controls [16]. In addition, our results demonstrate that upper FRT tissues contain cells that are highly susceptible to HIV-1 fusion, supporting the hypothesis that the cervical transformation zone and the endometrium are potential sites of HIV-1 acquisition. The fact that our results for the copper IUD are congruent with recent results from a randomized trial in women [10] is reassuring that the HIV-1 fusion assay may have relevance for predicting contraceptive safety, suggesting that HIV-1 risk should not be increased in IUD users and women on COCs compared to those not on these forms of contraceptives.

## Supporting information

S1 Fig Effect of contraceptives on HIV-1 acquisition as measured by prevalence of fusion susceptibility of endometrial cells to HIV-1 fusion *ex vivo* mediated by three different viral clones. Single cell suspension from EMBs were infected for 1.5h with M247Fv2, REJO and RHPA (500 ng p24^Gag^). Bar graphs depict the mean prevalence of immune cells supporting ZM247v2 fusion (overall height of the bar, with confidence limits) by donor contraception group. Contributions of four major cellular subsets to the overall prevalence are identified by color clade. OCP is combined oral contraceptive.
(TIFF)
