# Supplementary figures and images for: Effects of the levonorgestrel-containing intrauterine device, copper intrauterine device, and levonorgestrel-containing oral contraceptive on susceptibility of immune cells from cervix, endometrium and blood to HIV-1 fusion measured ex vivo
Source: PLoS One. 2019 Aug 22;14(8):e0221181. doi: 10.1371/journal.pone.0221181 (PMC6705759; doi:10.1371/journal.pone.0221181)

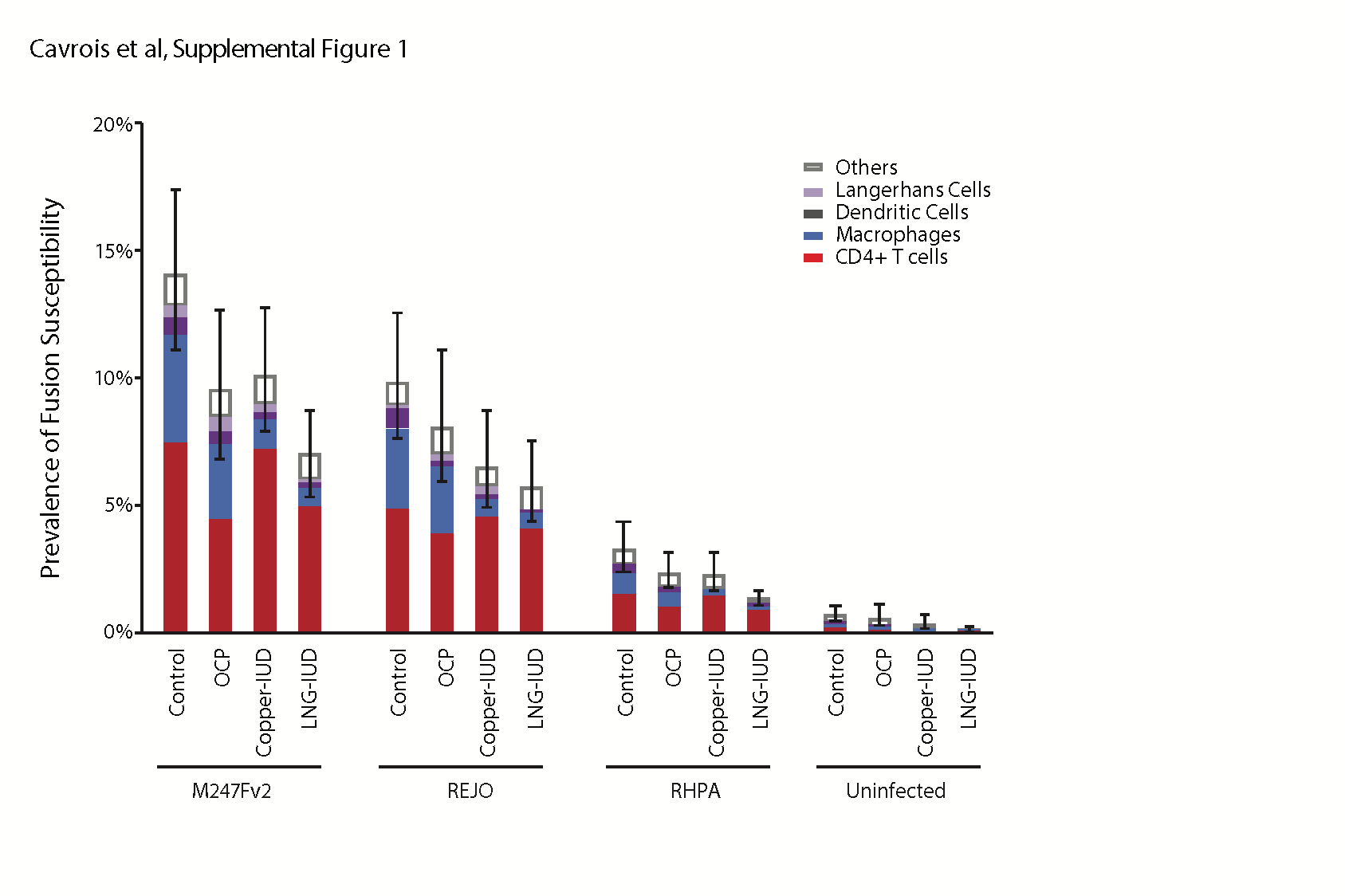

Supplement: S1 Fig — Single cell suspension from EMBs were infected for 1.5h with M247Fv2, REJO and RHPA (500 ng p24Gag). Bar graphs depict the mean prevalence of immune cells supporting ZM247v2 fusion (overall height of the bar, with confidence limits) by donor contraception group. Contributions of four major cellular subsets to the overall prevalence are identified by color clade. OCP is combined oral contraceptive. (TIFF) [file pone.0221181.s001.tiff]
